# A rare presentation of a gastric volvulus in an adult Bochdalek hernia: a case report

**DOI:** 10.1093/jscr/rjad540

**Published:** 2023-09-29

**Authors:** Anith Azhar, Zeti R A Karim, Yahya M Aripin, Muhammad Abid Amir

**Affiliations:** Department of Cardiovascular and Thoracic Surgery, Faculty of Medicine, Universiti Teknologi MARA (UiTM), 47000, Sungai Buloh, Selangor, Malaysia; Department of Surgery, Faculty of Medicine, Universiti Teknologi MARA (UiTM), 47000, Sungai Buloh, Selangor, Malaysia; Department of Surgery, Faculty of Medicine, Universiti Teknologi MARA (UiTM), 47000, Sungai Buloh, Selangor, Malaysia; Department of Cardiovascular and Thoracic Surgery, Faculty of Medicine, Universiti Teknologi MARA (UiTM), 47000, Sungai Buloh, Selangor, Malaysia

**Keywords:** gastric volvulus, Bochdalek hernia, diaphragmatic defect

## Abstract

A Bochdalek hernia is a common congenital diaphragmatic defect in infants. A late presentation during adulthood is rare with misleading signs and symptoms, resulting in misdiagnosis and errors in treatment. We describe a 30-year-old man who presented with abdominal pain and chronic choking sensation, which was previously treated as peptic ulcer disease. During the present admission, radiological imaging performed revealed loops of bowel and a gastric volvulus in the left hemithorax. The patient underwent a successful emergency surgery and repair of a Bochdalek hernia. Due to its rarity and ambiguous presentation, a symptomatic Bochdalek hernia in an adult is commonly misdiagnosed. A comprehensive evaluation is pertinent for early diagnosis and treatment, to prevent complications arising from obstruction and strangulation of herniated intraabdominal contents.

## Introduction

A Bochdalek hernia is a transposition of abdominal organs into the thoracic cavity through a congenital defect in the closure of the pleuroperitoneal canal, located between the lateral (costal) and posterior (crural) components of the diaphragm. Most cases occur in the neonatal and postnatal period. Although it can present late during adulthood, this is extremely rare and is commonly misdiagnosed [1]. Adult Bochdalek hernia usually presents with chronic respiratory or gastrointestinal symptoms such as dypsnoea, chest pain, abdominal pain, nausea, vomiting, and postprandial fullness. In asymptomatic patients, the diagnosis is usually incidental when abdominal organs are found in the thorax on imaging. However as in our case report, the diagnosis in symptomatic patients is made more challenging due to the non-specific nature of the symptoms.

## Case report

**Figure 1:**
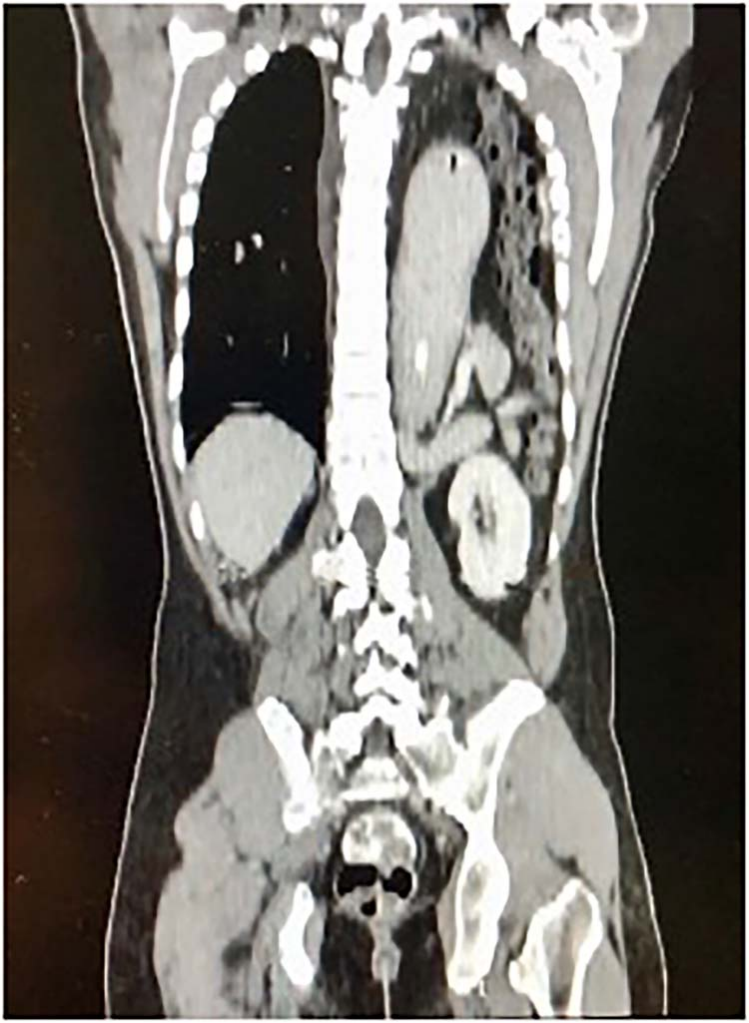
Longitudinal thoraco-abdominal CT scan shows protrusion of stomach, small and large bowel through a left posterolateral hemi-diaphragmatic defect.

**Figure 2:**
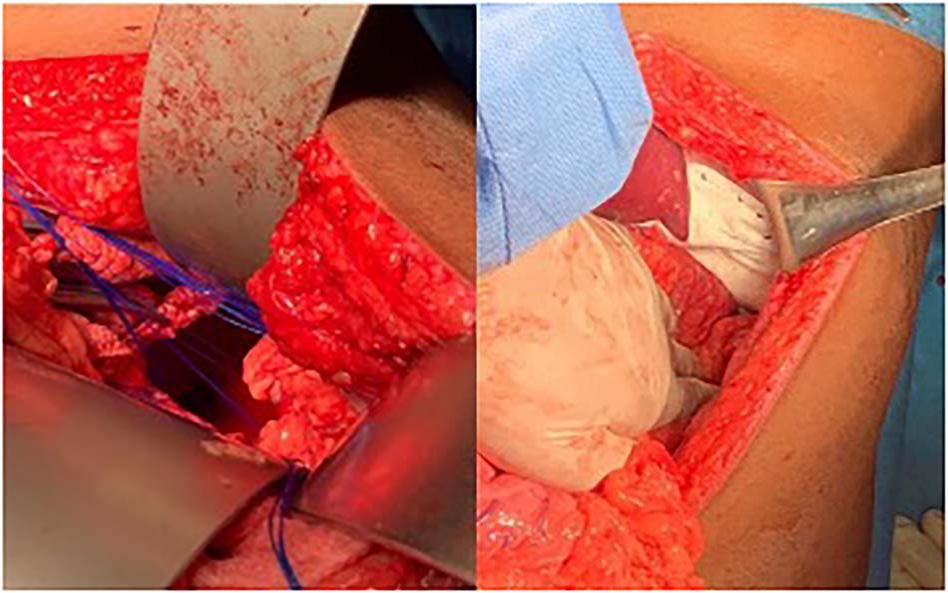
Left Photograph showing a posterolateral defect at the left hemi-diaphragm. Right Photograph showing defect repaired using composite mesh.

A 30-year-old male patient presented with a 1-week history of intermittent fever, generalized abdominal pain, vomiting, and coughing. He had been complaining of multiple episodes of choking sensation for several years which was treated as peptic ulcer disease. His past medical and surgical history were unremarkable with no previous history of trauma. On examination, he was tachycardic with a low grade fever. There was reduced left sided chest expansion with reduced breath sounds on auscultation. Interestingly, bowel sounds were heard over the left chest. On palpation of the abdomen, the left hypochondrium was tender and guarded. A Computed Tomography (CT) scan of the thorax and abdomen was done and showed a large left posterolateral diaphragmatic defect with herniation of the stomach, mesenteric fats, spleen, pancreatic tail, and bowel into the left hemithorax with mesenteroaxial gastric volvulus (Fig. 1). An exploratory laparotomy revealed a large posterolateral defect in the left hemi-diaphragm through which herniation of viable intraabdominal contents occurred. The contents were reduced into the intraabdominal cavity and the defect was repaired using composite mesh anchored with prolene 2/0 suture (Fig. 2). The patient made an uneventful post-operative recovery.

## Discussion

Bochdalek hernia was first described in 1848 by the Czechoslovakian anatomist, Vincent Alexander Bochdalek [1]. It is a posterolateral congenital defect of the diaphragm with 80–90% of cases occurring on the left [2]. The majority of cases occur in the neonatal and postnatal period. Although it can present late during adulthood, it is extremely rare with a prevalence of 0.17–6% of all diaphragmatic hernias and is commonly misdiagnosed due to its non-specific presentation [3].

As most Bochdalek hernia occurs on the left side, a large number presents with gastric herniation. Other organs involvement include colon, spleen, small, and large intestines. Increase in abdominal pressure such as chronic cough and choking can further aggravate the protrusion of the abdominal contents into the thoracic cavity with the occurrence of a gastric volvulus mainly due to laxity or absence of gastrohepatic, gastrocolic, gastrosplenic, and gastrophrenic ligaments [4–6].

Radiological imaging, particularly a CT scan of the thorax and abdomen, is important in the diagnosis and exclusion of other differentials, enabling the identification of a diaphragmatic defect and a continuous density over and under the diaphragm's discontinuity to suggest a diaphragmatic hernia [7].

The surgical approach depends on the presence of visceral complications. Most authors recommend the thoracic approach for elective cases; on the other hand, when there are complications, the abdominal approach is preferred. Minimally invasive techniques such as laparoscopy and thoracoscopy are gaining traction. In our case, an abdominal approach was employed because of the presence of a gastric volvulus and suspicion of peritonitis. A large diaphragmatic defect was visualized and in the absence of contamination, a decision was made for a mesh repair. Most defects can be closed primarily using non-absorbable sutures, however when a defect is large, a tension-free mesh is chosen to ensure a successful repair. As such, a primary closure would not have been possible in our case due to the size of the defect, necessitating the use of a mesh to provide a tension-free repair. Most patients with late-onset congenital diaphragmatic hernia (CDH) do well after surgical repair with apparently normal ipsilateral lung volume [8].

CDH is an uncommon diagnosis among the adult population due to its misleading and non-specific signs and symptoms. A thorough clinical examination and radiological imaging are important for an accurate diagnosis and exclude other differentials. The findings of a gastric or intestinal gas shadow in the chest cavity on a radiograph warrants a CT scan for better delineation as the knowledge of an anatomic defect and ensuing complications are crucial to dictate the correct surgical approach and planning. This case demonstrates a rare presentation of Bochdalek hernia in an adult patient complicated with a gastric volvulus. Clinicians need to be aware of this rare but life-threatening clinical entity in order to secure a timely diagnosis and institute appropriate management.

## Data Availability

The data that support the findings of this study are available on request from the corresponding author.
